# Influence of vitamin D receptor polymorphisms on biochemical markers of mineral bone disorders in South African patients with chronic kidney disease

**DOI:** 10.1186/s12882-018-0831-7

**Published:** 2018-02-07

**Authors:** Bala Waziri, Therese Dix-Peek, Caroline Dickens, Raquel Duarte, Saraladevi Naicker

**Affiliations:** Department of Internal Medicine, Faculty of Health Sciences, University of the Witwatersrand, Johannesburg, South Africa

**Keywords:** Chronic kidney disease, Secondary hyperparathyroidism, VDR polymorphisms

## Abstract

**Background:**

It remains unclear whether genetic factors may explain the reported variation in the levels of biochemical markers of chronic kidney disease mineral and bone disorders (CKD- MBD) across ethnic groups. Therefore, the aim of this study was to examine the influence of vitamin D receptor (VDR) polymorphisms on secondary hyperparathyroidism and its association with vitamin D levels in black and white South African study participants.

**Methods:**

This was a cross sectional study involving 272 CKD stage 3- 5D patients and 90 healthy controls. The four major VDR polymorphisms (*Bsm 1, Fok 1, Taq 1, and Apa1*) were genotyped using the polymerase chain reaction- restriction fragment length polymorphism (PCR –RFLP) method. In addition, biochemical markers of CKD-MBD were measured to determine their associations with the four VDR polymorphisms.

**Results:**

With the exception of *Taq I* polymorphism, the distribution of the VDR polymorphisms differed significantly between blacks and whites. In hemodialysis patients, the Bb genotype was significantly associated with moderate secondary hyperparathyroidism (OR, 3.88; 95 CI 1.13–13.25, *p* = 0.03) and severe hyperparathyroidism (OR, 2.54; 95 CI 1.08–5.96, *p* = 0.03). This was consistent with the observed higher levels of median parathyroid hormone, fibroblast growth factor 23 and mean phosphate in patients with Bb genotype. This candidate risk genotype (Bb) was over represented in blacks compared to whites (71.0% versus 55.6%, *p* < 0.0001). In an unadjusted regression model, *FokFf* genotype was found to be significantly associated with the risk of developing severe vitamin D deficiency < 15 ng/ml (OR, 1.89; 95 CI 1.17–3.07, *p* = 0.01).

**Conclusion:**

The VDR Bb genotype is an independent predictor of developing secondary hyperparathyroidism in patients with end stage kidney disease. In addition, study participants with *FokFf* genotype are at increased of developing severe 25 -hydroxyvitamin D [25(OH)D] deficiency.

## Background

Vitamin D deficiency has been linked to various disease conditions and poor clinical outcomes [1–3]. The widespread consequences of vitamin D deficiency have been partly attributed to the ubiquitous distribution of the vitamin D receptor [4]. The vitamin D receptor (VDR) plays a vital role in mediating the effects of the biologically active form of vitamin D (1, 25, OH-D); therefore it is plausible that variations in these receptors will modulate the consequences associated with vitamin D deficiency [5]. In 1994, Morrison et al. [6] were the first to report an association between VDR polymorphisms and bone metabolism, showing that the common allelic variants in the VDR encoding genes can predict differences in bone density in healthy individuals [6]. Subsequently, several researchers have explored this relationship in CKD populations with emphasis on the calcium/ PTH/ calcitriol axis [7, 8]. *BsmI* polymorphism (BB genotype) has been associated with slower progression of secondary hyperparathyroidism and normal levels of calcitriol in pre dialysis CKD patients, and lower levels of parathyroid hormone (PTH) in hemodialysis, and a greater reduction in PTH levels in response to a single bolus of calcitriol therapy compared to patients with bb genotype [8, 9]. However, contrary to earlier studies, findings from subsequent studies on the associations between VDR polymorphisms and markers of mineral bone disease have been inconsistent. For instance, some studies reported no difference in PTH levels between the various *Bsm I* genotypes [10, 11], while Chudek et al. reported significantly lower levels of calcitriol in patients with BB genotype [12]. Similarly, some studies have linked other VDR polymorphisms to mineral bone metabolism in hemodialysis patients. The VDR *Fok I* polymorphism (FF genotype) was reported to be associated with higher PTH levels [13].

Furthermore, the existence of racial disparities in abnormal markers of CKD-MBD and the better survival paradox in African Americans compared to white dialysis patients may be explained partly by the racial differences in the distribution of VDR polymorphisms and VDR receptor activation therapy. Most of these studies were conducted on European, Asian and American CKD populations, while studies from Africa were largely on non CKD populations. Therefore, in line with ongoing efforts to greater understanding of the mechanisms behind racial disparities in markers of CKD- MBD, we aimed to explore the variations in the VDR polymorphisms between black and white African CKD patients and their relationship with markers of mineral bone disorders.

## Methods

This was a cross sectional study involving 272 CKD stage 3-5D patients and 90 healthy controls. The study protocol was approved by the Health Research and Ethics committee (HREC) of the University of the Witwatersrand; clearance certificate number M141016. All participants gave written informed consent prior to enrolment. Exclusion criteria included active malignancies, primary hyperparathyroidism, genetic related calcium disorders such as autosomal dominant hypercalciuric hypocalcemia, use of bisphosphonates, aged < 18 years, and patients who withheld consent.

Information on participants’ demographic characteristics, duration on dialysis and use of medications related to CKD-MBD were obtained. Determination of race was based on self report by the participants.

### Laboratory measurements

Plasma intact PTH was measured by an electrochemiluminescence immunoassay (ECLIA) run on a Cobas 6000 auto analyzer (Roche Diagnostics, Mannheim, Germany).

FGF23 was measured using an enzyme-linked immunosorbent assay kit from EMD Millipore Corporation (Billerica, MA, USA). Assay lower detect limit was 3.2 pg/ml.

Plasma 25(OH) D was measured using the high performance liquid chromatography (HPLC) kit (Recipe, Munich, Germany). HPLC was used to selectively measure 25(OH)D2 and 25 (OH)D3 at a wave length of 264 nm. The intra and inter assay coefficients of variation (CVs) were < 5%. Our institutional laboratory is a participating member in the vitamin D external quality assurance scheme (DEQAS).

Serum calcium, phosphate and alkaline phosphatase were measured using the ADVIA 1800 centaur auto analyzer (Siemens Diagnostics, Tarrytown, USA). The albumin corrected serum calcium was determined using the formula: corrected calcium (mmol/L) = calcium measured (mmol/L) + 0.02 [40-albumin (g/L)].

Serum creatinine was measured by a modified Jaffe reaction and GFR was estimated using the four- variable Modified Diet Renal Disease (MDRD) equation [14]: GFR (in mL/min per 1.73 m^2^) = 175 × SCr (exp[− 1.154]) × Age (exp[− 0.203]) × (0.742 if female) × (1.21 if black).

Other biochemical parameters were determined using routine laboratory techniques.

### Genotyping

DNA was extracted from whole blood using the Maxwell DNA purification kit (Promega AS1010, USA). Using appropriate primers obtained DNA products were amplified for *ApaI* (Foward: 5’ CAGAGCATGGACAGGGAGCAAG 3′ and Reverse: 5’ GCAACTCCTCATGGCTGAGGTCTCA 3′ with 65 °C annealing temperature), *BsmI* (Forward: 5’ CAACCAAGACTACAAGTACCGCGTCAGTGA 3′ and Reverse: 5’ AACCAGCGGGAAGAGGTCAAGGG 3 ‘with 65 °C as an annealing temperature), *FokI* (Forward: 5’ AGCTGGCCCTGGCACTGACTCTTGCTCT 3′ and Reverse: 5’ ATGGAAACACCTTGCTTCTTCTCCCTC 3′ with 67 °C annealing temperature), and *TaqI* (Forward: 5’ CAGAGCATGGACAGGGAGCAAG 3′ and Reverse: 5’GCAACTCCTCATGGCTGAGGTCTCA 3′ at an annealing temperature of 65 °C) VDR polymorphisms. The PCR products were then digested with enzymes *ApaI, BsmI, FokI,* and *TaqI* (New England Biolabs, Beverly, MA, USA) according to the supplier’s protocol. Digestions for *BsmI and TaqI* were at 65 °C left overnight, and 3 h at 25 °C for *ApaI*, while *FokI* was incubated at 37 °C for 3 h. Restricted products were electrophoresed on either 10% polyacrylamide or 1.5% agarose gels and then visualized by the Gel Doc TM EZ imager (Bio-Rad systems, USA). Genotypes were scored based on the presence or absence of a restriction site for the enzymes *BsmI, ApaI, and TaqI* at the 3′ untranslated region and *FokI* at the N-terminal region of the gene.

### Statistical analysis

The Fisher’s exact test was utilized to compare differences in the frequency of genotypic distribution between groups. Based on the distribution of data, an independent t – test or Wilcoxon rank –sum test were used to compare continuous variables between two groups, while one- way ANOVA or Kruskal –Wallis tests were used for more than two groups. Both univariate and multivariable logistic regression models were used to determine the association between VDR genotypes, secondary hyperparathyroidism and vitamin D deficiency. A backward selection procedure was used to fit the multiple regression model, which started with all potential predictor variables and subsequently removed the variables that had *p* values above the specified *P* = 0.20. However, variables that are known to be biologically plausibly associated with secondary hyperparathyroidism were forced into the model despite not meeting inclusion criteria based on the stepwise approach. A post estimation test for Goodness of Fit of the models was carried out using Hosmer -Lemeshow goodness of fit test.

In the comparisons of the means and medians of the circulating markers of CKD-MBD across the genotypes, the *P* values for distribution between homozygous dominant and heterozygous genotypes were further determined separately due to the smaller numbers of the homozygous recessive.

A *p*-value of less 0.05 was considered statistically significant at the 95% confidence interval. All analyses were performed using STATA version 12 (STATA Corp., TX, and USA).

## Results

### Description of the study population

**Fig. 1:**
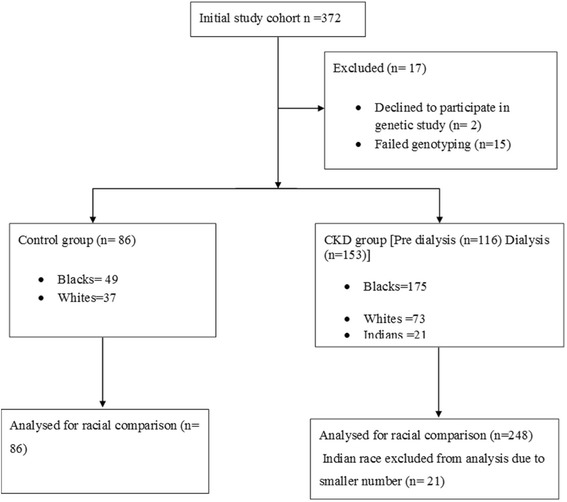
Participant disposition and recruitment flow chart

A total of 362 participants (272 CKD patients and 90 controls) were recruited for this study. The CKD group comprised 156 CKD stage 5D and 116 CKD stages 3–5 patients. In the control group, 39 participants were self-identified as whites, 60 as blacks, and one Indian. The CKD group comprised 73 whites, 175 blacks and 21 Indians. Fifteen patients were excluded from the genetic analysis due to failed genotyping (Fig. 1). Patients on haemodialysis were on three times weekly, 4 h sessions of haemodialysis using polysulphone membranes and bicarbonate dialysate. Most of the patients were dialyzed with a dialysate calcium concentration of 1.50 mmol/L, which is usually modified based on serum levels of calcium. The blood and dialysis flow rates are generally 300–400 mls/min and 500 mls/min, respectively, as previously reported [15].

Table 1 shows the study participants characteristics and ethnic distribution of the VDR polymorphisms (*Bsm I, FokI, ApaI and Taq I*). In the VDR polymorphisms, blacks had significantly higher proportion of Bb genotype than whites (71.0%versus 55.6%), and lower frequency of BB genotype (24.1% versus 44.4%). Overall, the most common genotype was Bb (65.4%). Similarly, the distribution in *FokI andTaq I* genotypes differed significantly between the groups; FF was more frequent in blacks, while Ff genotype was the most prevalent in whites. There was no significant ethnic variation in the distribution of the *ApaI* genotype. With the exception of *Apa I* genotype, the genotype distributions did not violate Hardy-Weinberg equilibrium.

**Table 1:** Participants characteristics and genotype frequencies by race

Parameters	Black (*n* = 224)	White (*n* = 110)	P-value
Age (years)	46.5 ± 12.9	54.4 ± 17.5	< 0.0001
Gender n (%)			
Male	111 (49.6%)	60 (54.5%)	0.74
Female	113 (50.4%)	50 (45.5%)	
25- O HD (ng/ml)	25.8 ± 12.1	23.1 ± 11.9	0.048
PTH (pg/ml)	214 (61–872)	112 (30–364)	0.001
Calcium (mmol/l)	2.22 ± 0.25	2.29 ± 0.18	0.06
TAP (U/L)	120 (88–190)	110 (74–145)	0.14
Phosphate (mmol/l)	1.29 ± 0.47	1.48 ± 0.49	0.003
FGF 23 (ng/ml)	59 (23–307)	80 (28–521)	0.20
VDR genotypes			
*Bsm I*			
BB	54 (24.1%)	48 (43.6%)	P < 0.0001
Bb	159 (71.0%)	62 (56.4%)	
bb	11 (4.9%)	0 (0.00%)	
*Fok I*			
FF	151 (67.4%)	38 (34.6%)	P < 0.0001
Ff	71 (31.7%)	69 (62.7%)	
ff	2 (0.89%)	3 (2.73%)	
*Apa I*			
AA	94 (42.0%)	40 (36.4%)	0.61
Aa	127 (56.7%)	69 (62.7%)	
aa	3 (1.34%)	1 (0.91%)	
*Taq I*			
TT	128 (57.1%)	44 (40.0%)	0.01
Tt	80 (35.7%)	52 (47.3%)	
tt	16 (7.1%)	14 (12.7%)	

Table 2 shows the distribution of the four VDR polymorphisms (*Bsm I, FokI, ApaI and Taq I*) between CKD patients and healthy controls, and the odds ratio of developing severe 25 (OH) D severe deficiency (< 15 ng/ml). The frequencies of these genotypes did not differ significantly between the CKD and control groups. Ff genotype showed a significant increase in odds of developing severe 25 (OH) D deficiency (OR, 1.89; 95 CI 1.17–3.07, *p* = 0.01); a similar trend was found with combined Ff + ff genotypes (OR, 1.91; 95 CI 1.18–3.08, *p* = 0.008). The remaining genotypes were not significantly associated with severe 25 (OH) D deficiency.

**Table 2:** Distribution of VDR polymorphisms among CKD and control groups and the odds ratios for developing severe 25 -hydroxyvitamin D severe deficiency (< 15 ng/ml)

VDR genotypes	Controls	*CKD*	p-value^a^	OR (95%CI)	P-value
*Bsm I*	*N* = 84	*N = 268*			
BB	23 (27.4%)	87 (32.5%)	0.05	1.00 (reference)	
Bb	55 (65.5%)	176 (65.7%)		0.85 (0.51–1.42)	0.55
bb	6 (7.14%)	5 (1.87%)		0.28 (0.03–2.32)	0.24
Dominant model					
BB	23 (27.4%)	87 (32.5%)			
Bb + bb	61 (72.6%)	181 (67.5%)	0.38	0.83 (0.50–1.37)	0.46
*Fok I*	*N* = 86	*N* = 266			
FF	45 (52.3%)	152 (57.1%)	0.47	1.00 (reference)	
Ff	39 (45.4%)	111 (41.7%)		1.89 (1.17–3.07)	0.01
ff	2 (2.3%)	3 (1.1%)		2.52 (0.41–15.59)	0.32
Dominant model					
FF	45 (52.3%)	152 (57.1%)	0.43	1.00 (reference)	
Ff + ff	41 (47.7%)	114 (42.9%)		1.91 (1.18–3.08)	0.008
*Apa I*	*N* = 83	*N* = 269			
AA	32 (38.6%)	112 (41.6%)	0.50	1.00 (reference)	
Aa	51 (61.4%)	152 (56.5%)		1.44 (0.88–2.37)	0.15
aa	0 (0.0%)	5 (1.86%)		2.33 (0.37–14.57)	0.37
Dominant model					
AA	32 (38.6%)	112 (41.6%)	0.70	1.00 (reference	
Aa +aa	51 (61.4%)	157 (59.0%)		1.46 (0.89–2.40)	0.13
*Taq I*	N = 84	N = 268			
TT	37 (44.1%)	146 (54.5%)	0.05	1.00 (reference)	
Tt	42 (50.0%)	95 (35.5%)		1.00 (0.61–1.65)	0.99
tt	5 (6.0%)	27 (10.1%)		0.76 (0.31–1.87)	0.60
Dominant model					
TT	37 (44.1%)	146 (54.5%)	0.06		
Tt + tt	47 (56.0%)	122 (45.5%)		0.96 (0.59–1.53)	0.85

*OR* odds ratio, *CI* confidence interval, *CKD* chronic kidney disease, p-value^a^ for comparison of genotype frequencies between control and CKD groups, VDR = vitamin D receptor

The biochemical markers of CKD-MBD in the various genotypes are shown in Table 3. Median PTH, FGF 23and mean phosphate levels were significantly higher in patients with Bb genotype. Although the mean weekly dose of alfacalcidol was comparable between BB and Bb genotypes, the proportion of patients on alfacalcidol was higher in patients with Bb genotypes (45.5 versus 32.2, *p* = 0.047). In a restricted comparison between homozygous dominant genotype and heterozygous genotype due to smaller numbers of homozygous recessive genotype, the *P* values did not change significantly.

**Table 3:** Levels of markers of CKD-MBD across various VDR genotypes

Variable	Genotypes			p-value	P-value^c^
*Bsm I*	BB (*n* = 87)	Bb (*n* = 176)	bb (*n* = 5)		
25-OH D (ng/ml)	21 (14–33)	25 (16–34)	27 (19–31)	0.30	0.14
PTH (pg/ml)	231 (111–593)	553 (197–1230)	169 (134–214)	< 0.001	< 0.001
Calcium (mmol/l)	2.24 ± 0.21	2.21 ± 0.26	2.15 ± 0.25	0.49	0.32
Corrected ca (mmol/l)	2.20 ± 0.75	2.21 ± 0.64	2.20 ± 0.10	0.99	0.98
Phosphate (mmol/l)	1.27 ± 0.49	1.43 ± 0.49	1.0 ± 0.33	0.002	0.01
TAP (U/L)	121 (83–153)	113 (88–173)	80 (57–141)	0.373	0.91
FGF 23	95 (34–414)	157 (52–1191)	33 (22–35)	0.047	0.08
Medications					
Calcium carbonate	37 (42.5)	89 (50.6)	2 (40.0)	0.19	0.07
Alfacalcidol	28 (32.2)	80 (45.5)	1 (20.0)	0.09	0.047
Caco3 (mg/day)	3643 ± 802	3577 ± 744	3750 ± 1060	0.94	0.84
Alfacalcidol (μg/week)	2.25 ± 1.22	1.81 ± 1.07	–	–	0.38
*Fok I*	FF (*n* = 152)	Ff (*n* = 111)	ff (*n* = 3)		
25-OH D (ng/ml)	24 (14–34)	22 (14–33)	15 (11–29)	0.31	0.30
PTH (pg/ml)	327 (121–975)	360 (166–735)	61 (28–94)	0.12	0.86
Calcium (mmol/l)	2.21 ± 0.27	2.23 ± 0.20	2.17 ± 0.43	0.78	0.55
Corrected ca (mmol/l)	2.21 ± 0.61	2.19 ± 0.76	2.17 ± 0.27	0.97	0.83
Phosphate (mmol/l)	1.35 ± 0.51	1.38 ± 0.48	1.61 ± 0.51	0.64	0.64
TAP (U/L)	123 (91–160)	103 (79–167)	258 (69–312)	0.47	0.35
FGF 23	105 (35–845)	161 (54–1109)	81 (22–130)	0.36	0.29
Medications					
Calcium carbonate	65 (42.8)	59 (44.1)	1 (33.3)	0.23	0.11
Alfacalcidol	60 (39.5)	45 (40.5)	1 (33.3)	0.91	0.74
Calcium carbonate (mg/day)	3545 ± 739	3692 ± 778	–	–	0.58
Alfacalcidol (μg/week)	1.98 ± 1.12	1.93 ± 1.12	–	–	0.91
*Taq I*	TT (*n* = 146)	Tt (*n* = 95)	tt (*n* = 27)		
25- OHD (ng/ml)	23 (15–32)	25 (16–36)	21 (15–32)	0.31	0.23
PTH(pg/ml)	363 (174–926)	327 (109–913)	672 (121–1314)	0.24	0.17
Calcium	2.22 ± 0.23	2.22 ± 0.25	2.26 ± 0.28	0.74	0.87
Corrected ca	2.21 ± 0.61	2.17 ± 0.82	2.36 ± 0.31	0.47	0.71
Phosphate (ng/ml)	1.31 ± 0.45	1.38 ± 0.56	1.52 ± 0.47	0.13	0.36
TAP (U/L)	110 (82–154)	123 (93–192)	121 (75–167)	0.39	0.17
FGF 23	100 (39–867)	150 (35–511)	263 (98–3834)	0.055	0.91
Medications					
Calcium carbonate	61 (41.8)	52 (54.7)	14 (51.9)	0.41	0.18
Alfacalcidol	54 (37.0)	43 (45.3)	11 (40.7)	0.50	0.24
Calcium carbonate (mg/day)	3474 ± 716	3900 ± 775	3500 ± 775	0.33	0.15
Alfacalcidol (μg/week)	1.68 ± 0.94	2.44 ± 1.32	2.25 ± 1.06	0.23	0.10
*Apa I*	AA (*n* = 112)	Aa (n = 152)	aa (n = 5)		
25 -OHD (ng/ml)	24 (17–37)	22 (15–32)	19 (15–28)	0.22	
PTH (pg/ml)	329 (137–957)	383 (99–814)	1889 (1359–1889)	0.04	0.63
Phosphate (mmol/l)	1.34 ± 0.52	1.37 ± 0.48	1.63 ± 0.47	0.46	0.52
Calcium	2.23 ± 0.25	2.21 ± 0.24	2.38 ± 0.14	0.27	0.52
Corrected ca	2.22 ± 0.66	2.19 ± 0.68	2.41 ± 0.16	0.79	0.76
TAP (U/L)	123 (82–190)	115 (88–149	160 (91–440)	0.51	0.91
FGF 23	109 (35–929)	121 (44–761)	335 (72–687)	0.69	0.65
Medications					
Calcium carbonate	49 (43.8)	74 (48.7)	5 (100.0)	0.04	0.23
Alfacalcidol	41 (36.6)	64 (42.1)	4 (80.0)	0.29	0.33
Calcium carbonate (mg/day)	3700 ± 775	3525 ± 734	3375 ± 750	0.68	0.50
Alfacalcidol (μg/week)	2.03 ± 1.06	1.93 ± 1.15	2.50 ± 0.87	0.71	0.83

Variables are presented as means± standard deviations or median(interquartile range), *TAP* serum total alkaline phosphate, *PTH* parathyroid hormone, *25 (OH)D* 25 hydroxyvitamin D 3, p-value^c^: comparison between Homozygous dominant and heterozygous genotypes

In a restricted analysis involving hemodialysis patients, the univariable and multivariable analyses for the odds of developing moderate (controlled) and severe (uncontrolled) secondary hyperparathyroidism are shown in Table 4. Moderate (controlled) secondary hyperparathyroidism was defined as PTH between 130 pg/ml and 585 pg/ml (2–9 times the upper limit of normal) and severe (uncontrolled) secondary hyperparathyroidism as PTH > 585 pg/ml (9 times the upper limit of normal). After adjusting for serum calcium, phosphate, fibroblast growth factor 23, and use of alfacalcidol, the Bb genotype was a significant predictor of developing both moderate (OR,3.88; 95 CI 1.13–13.25, *p* = 0.03) and severe hyperparathyroidism(OR, 2.54; 95 CI 1.08–5.96, *p* = 0.03). The use of alfacalcidol was not eligible for inclusion into the final model, but was forced into the model due to a biologically plausible association between secondary hyperparathyroidism and the use of alfacalcidol.

**Table 4:** Odds ratios for association between VDR polymorphisms and secondary hyperparathyroidism in haemodialysis patients

Polymorphisms	Crude OR 95% (CI)	P-value	Adjusted^a^OR 95% (CI)	P-value
Moderate (controlled) secondary hyperparathyroidism (PTH > 130 ng/ml)				
*Bsm I*				
BB	1.00 (reference)		1.00 (reference)	
Bb	3.12 (1.11–8.83)	0.03	3.88 (1.13–13.25)	0.03
bb	N/A	N/A	N/A	
*FokI*				
FF	1.00 (reference)		1.00 (reference)	
Ff	0.87 (0.31–2.39)	0.78	0.65 (0.20–2.10)	0.47
ff	N/A	N/A	N/A	
*Taq I*				
TT	1.00 (reference)		1.00 (reference)	
Tt	0.27 (0.09–0.84)	0.02	0.43 (0.12–1.52)	0.19
tt	0.53 (0.09–2.96)	0.47	0.76 (0.11–5.19)	0.78
*Apa I*				
AA	1.00 (reference)		1.00 (reference)	
Aa	0.42 (0.13–1.36)	0.15	0.25 (0.06–1.01)	0.052
aa	0.28 (0.3–3.14)	0.30	0.25 (0.01–3.10)	0.25
Severe (uncontrolled) hyperparathyroidism (PTH > 585 pg/ml)				
*Bsm I*				
BB	1.00 (reference)		1.00 (reference)	
Bb	2.55 (1.19–5.47)	0.02	2.54 (1.08–5.96)	0.032
bb	N/A	N/A	N/A	N/A
*Fok I*				
FF	1.00 (reference)		1.00 (reference)	
Ff	0.42 (0.21–0.82)	0.01	0.37 (0.17–0.81)	0.01
ff	N/A	N/A	N/A	
*Taq I*				
TT	1.00 (reference)		1.00 (reference)	
Tt	0.64 (0.31–1.32)	0.23	0.71 (0.32–1.59)	0.41
tt	1.37 (0.44–4.24)	0.58	1.39 (0.41–4.73)	0.39
*Apa I*				
AA	1.00 (reference)		1.00 (reference)	
Aa	0.85 (0.43–1.69)	0.65	0.74 (0.35–1.57)	0.43
aa	2.26 (0.24–21.47)	0.48	2.84 (0.27–30.22)	0.86

*OR* odds ratio, *CI* confidence interval, *N\A* not applicable

^a^Adjusted Odd ratio = adjusted for Age, calcium, phosphate, Fibroblast growth factor 23 and use of alfacalcidol

The post estimation test shows no lack of fit with the final models (*p* > 0.05).

**Fig. 2:**
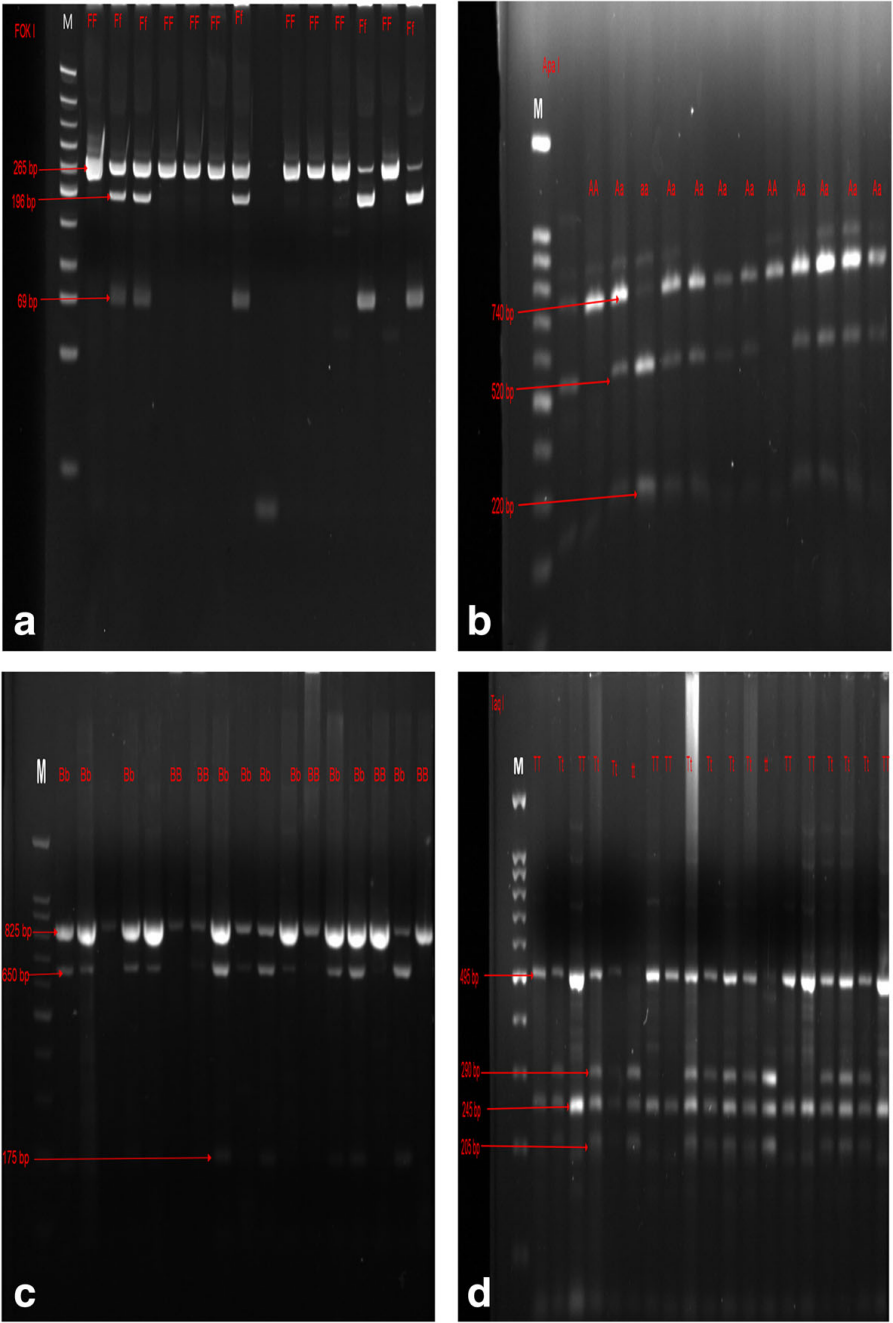
**a**-**d** Restriction endonuclease digestion for *FokI, ApaI, Bsm I and TaqI* polymorphisms, M = Marker

Figure 2a-d show gels for restriction endonuclease digestion for *FokI*, *Apa I, Bsm I and Taq I* polymorphisms respectively.

## Discussion

In an attempt to unravel the complexity behind the pathophysiologic mechanisms of CKD-MBD, several investigators have looked at the relationship between VDR polymorphisms and the calcium/PTH/calcitriol axis with inconsistent findings [5, 12]. In this present study, consistent with some previous reports, we found a significant difference in PTH levels across *Bsm I* genotypes, patients with Bb genotype had a higher median PTH level compared to patients with BB and bb genotypes. In addition, Bb genotype was independently associated with the risk of developing moderate and severe secondary hyperparathyroidism in patients with ESKD. The influence of *BsmI* on parathyroid function was also observed in pre dialysis CKD and transplant patients. Marco et al. [16] reported a slower progression of secondary hyperparathyroidism in pre dialysis CKD patients with BB genotype, while Messa et al. [17] reported lower PTH levels in transplant patients with BB genotypes. On the other hand, contrary to our findings, some studies have reported non-significant differences in PTH levels across *BsmI* genotypes. However, it is noteworthy that *Bsm I* genotypes distribution varies greatly across ethnic groups, hampering comparisons of studies.

The molecular mechanisms by which *Bsm I* VDR polymorphisms influence hyperparathyroidism have been linked to presence of b alleles. Previous studies have reported a strong association between b alleles and decreased VDR gene transcription and/or m RNA stability, hence, affecting the regulatory actions of calcitriol on parathyroid glands [6, 17]. For example, patients with the BB genotypes are less susceptible to having reduced 1α-hydroxylase compared to patients with bb genotypes. Therefore, patients with b alleles are less likely to have optimal levels of calcitriol required to inhibit PTH secretion and parathyroid cell proliferation.

A few studies have also investigated the associations between *FokI, ApaI* and secondary hyperparathyroidism in patients with CKD. Consistent with a previous study [13], although not statistically significant, patients with FF genotype had lower levels of PTH than patients with Ff in our study.

In addition to the complexity of CKD -MBD is the existence of the ethnic variability in the development and severity of secondary hyperparathyroidism among CKD patients. Several previous studies consistently showed that black patients have higher PTH levels and lower 25 OH vitamin D levels [18, 19]. The mechanisms behind these dissimilarities may partly be explained by genetic factors. For example, some polymorphisms may be over represented in certain races and therefore alter their risk. In this present study, there was a statistically significant difference between black and white participants in the distribution of the VDR polymorphisms. The Bb genotype which is an independent predictor of hyperparathyroidism is over expressed in black populations (71.0% versus 56.4%, *p* < 0.0001). In line with our findings, previous studies have also reported ethnic variations in the distribution of VDR polymorphisms [20, 21]. Uitterlinden et al. [21] reported that the frequency of the f allele of *Fok1* was lower in Africans as compared to Caucasians (Caucasians 34% versus Africans 24%); similarly a significant difference was found in the frequency of the *Bsm1*, B allele was lower in the Asian population compared to other populations (Asians 7%, Africans 36%, and 42% in Caucasians). These observed ethnic variations in the frequency of the VDR polymorphisms may help in explaining the racial discrepancy in the markers of CKD-MBD.

Several studies have consistently associated vitamin D insufficiency to various skeletal and extra skeletal clinical end points, leading to a special interest in the determinants of vitamin D metabolites [21, 22]. Thus far, well-established determinants of 25(OH) Dlevels include dietary sources and sun exposure [22]. However, a genetic factor has been shown to play a vital role in the inter individual variation in circulating vitamin D levels. For example, in the classical twin study, Hunter et al. reported that the calcium/PTH/calcitiol axis is under strong genetic influence, accounting for 52% of calcium excretion, 74% of bone formation, 58% of bone resorption, 60% of PTH, and 65% of vitamin D variance [23]. Similarly, a more recent large GWAS study has revealed a significant association with some genetic variants with 25(OH) D levels [22]. These important findings were restricted to Caucasians, limiting their results to other ethnic groups. However, a few studies that explored these associations across races yielded similar results [24]. In agreement with these studies, we found an increased risk of developing severe vitamin D deficiency with *Fok* Ff genotype and combined Ff + ff genotypes. In contrast, we did not find a significant difference in vitamin D levels across the various VDR genotypes.

The limitations of our study include the following. Firstly, the influence of some wild type genotype (homozygous minor) on the calcium/PTH/calcitiol axis could not be adequately determined due to their smaller numbers. Thus, a larger sample will be required to detect their associations with markers of CKD -MBD. Secondly, this was a cross-sectional study design; therefore we could not determine the longitudinal changes in markers of CKD- MBD, as well as seasonal variation in 25 (OH) D levels. Thirdly, Information relating to UVB exposure and vitamin D dietary history are lacking.

The strength of this study lies in the heterogeneous nature of our study population (black and white patients) in an African setting which has allowed comparisons of data not only for Black Africans with Black Americans, but also between whites in Africa and USA/Europe.

## Conclusion

We have demonstrated that both moderate and severe secondary hyperparathyroidism are predicted by *BsmI* Bb genotype, and the over expression of this genotype in black patients may partly explain the ethnic variations in the severity of secondary hyperparathyroidism in CKD population. In addition,* Fok *Ff genotype might be an important determinant of an individual’s susceptibility to 25 (OH)D deficiency.

## Abbreviations

25(OH) D: 25- hydroxyvitamin D
CKD- MBD: Chronic kidney disease- mineral and bone disorder
CKD: Chronic kidney disease
DEQAS: Vitamin D external quality assurance scheme
DNA: Deoxyribonucleic acid
ECLIA: Electrochemiluminescence immunoassay
EDTA: Ethylene diamine tetra acetic
eGFR: Estimated glomerular filtration rate
ESKD: End stage kidney disease
FGF 23: Fibroblast Growth Factor 23
HPLC: High performance liquid chromatography
iPTH: Intact Parathyroid hormone
MDRD: Modified Diet Renal Disease
MHD: Maintenance haemodialysis
OR: Odds ratio
VDR: Vitamin D receptor
